# Substance-Based Medical Device in Wound Care: Bridging Regulatory Clarity and Therapeutic Innovation

**DOI:** 10.3390/polym18010129

**Published:** 2025-12-31

**Authors:** Daiana Ianev, Michela Mori, Barbara Vigani, Caterina Valentino, Marco Ruggeri, Giuseppina Sandri, Silvia Rossi

**Affiliations:** 1 Department of Drug Sciences, University of Pavia, Viale Taramelli 12, 27100 Pavia, Italy; daiana.ianev01@universitadipavia.it (D.I.); barbara.vigani@unipv.it (B.V.); caterina.valentino@unipv.it (C.V.); marco.ruggeri@unipv.it (M.R.); giuseppina.sandri@unipv.it (G.S.); 2 1Med SA, Via Campagna 13, 6982 Agno, Switzerland

**Keywords:** substance-based medical device, medical device made of substance, topical, medicinal products, skin

## Abstract

Substance-based medical devices (SBMDs) are increasingly used in wound care due to their favorable safety profile, physicochemical mechanisms of action, and therapeutic effectiveness. These products often incorporate biopolymers such as hyaluronic acid or chitosan, alone or in combination with antimicrobial agents like silver nanoparticles (AgNPs) or silver sulfadiazine (SSD), offering hydration, tissue protection, and control of microbial burden in both acute and chronic wounds. Despite their widespread clinical use, the regulatory classification of SBMDs under Regulation (EU) 2017/745 (MDR) remains one of the most challenging and debated areas within the current European framework. This review analyzes the scientific and regulatory context of topical SBMDs, with particular emphasis on borderline products that share similarities with medicinal products in terms of formulation, composition, or claimed effects. The discussion focuses on the application of MDR Annex VIII, specifically Rule 21 for substance-based devices and Rule 14 for devices incorporating medicinal substances with ancillary action, together with interpretative guidance provided by MDCG 2022-5 Rev.1 and the Association of the European Self-Care Industry (AESGP) Position Paper. Particular attention is given to the identification of the critical role of the primary mode of action (MoA) as the determining criterion for regulatory qualification, especially for products containing antimicrobial substances. Through selected examples and case analyses, the review highlights inconsistencies in classification across Member States and underscores the need for a more harmonized, evidence-based, and proportionate regulatory approach. Overall, SBMDs challenge traditional regulatory boundaries and call for a framework capable of accommodating complex, multifunctional products while ensuring patient safety and regulatory coherence.

## 1. Introduction

The application of topical products in healthcare has deep historical roots, originating from ancient civilizations that utilized natural extracts and botanicals for therapeutic, protective, and cosmetic purposes [1]. Throughout the 20th century, advancements in pharmaceutical science, dermatology, and formulation technology significantly transformed topical products, facilitating the introduction of sophisticated dosage forms such as creams, gels, ointments, and transdermal patches, designed to enhance bioavailability and controlled substance delivery [2]. Parallel to these technological advancements, the regulatory landscape also evolved to address the complexity and increasing overlap among medicinal products and medical devices, each governed by distinct European legislations: Directive 2001/83/EC for medicinal products and, more recently, the Medical Device Regulation (EU) 2017/745 (MDR) for medical devices. Medical devices (MDs) and medicinal products (MPs) constitute the essential therapeutic tools available in contemporary healthcare and are required to address the increasingly complex demands posed by both acute and chronic conditions. Although both regulatory frameworks aim to guarantee product safety and efficacy, they are based on fundamentally different principles and target distinct categories of products. The development of innovative health solutions is only achievable within an appropriate regulatory framework [3]. In this context, the qualification of certain products is not always straightforward, leading to extensive discussions at the European level regarding so-called borderline products. Due to their inherent characteristics, these products do not clearly fall within a specific regulatory category, making it challenging to determine the applicable legal framework [4].

The implementation of Regulation (EU) 2017/745 has significantly increased the complexity of product qualification at the interface between medical devices and medicinal products, making substance-based medical devices (SBMDs) a paradigmatic case of borderline classification under the MDR, where regulatory determination critically depends on the identification of the principal mode of action (MoA).

Among the most common borderline products are Integral Drug-Device Combinations (IDDCs), Drug-Device Combinations (DDCs), and Substance-Based Medical Devices (SBMDs). These categories, although distinct, often present classification challenges due to their overlapping characteristics with both MDs and MPs [2].

This review aims to provide a comprehensive overview of a specific category of borderline product: substance-based medical devices (SBMDs) for topical applications. It focuses on their regulatory definitions, classification criteria, and key distinguishing features in comparison to topical MPs. The review addresses their mechanisms of action, clinical applications, market landscape, and the current challenges associated with their classification under the European regulatory framework. Through selected examples and case studies, particular emphasis is placed on the application of MDR Annex VIII, Rules 14 and 21, in order to illustrate the practical implications for product development and regulatory compliance. Overall, topical SBMDs exemplify how formulation complexity and multifunctionality can amplify regulatory uncertainty, highlighting the need for a scientifically robust and consistent approach to classification under the MDR.

## 2. Substance-Based Medical Devices (SBMDs)

Under Regulation (EU) 2017/745, which became fully applicable in May 2021, the definition of a medical device is given in Chapter 1, Article 2. According to this definition, a product qualifies as a medical device if it meets two key conditions: (1) it must serve a specific medical purpose on a human being, and (2) its principal intended action must not be achieved by pharmacological, immunological or metabolic (PhIM) means. Essentially, any tool or apparatus used in medical practice in humans that achieves its intended purpose neither as a medicinal product nor as a nutritional product (such as parenteral nutrition) can be classified as a medical device [5,6]. In this regard, the MDCG 2022-5 Rev. 1—“*Guidance on borderline between medical devices and medicinal products”* gives the definitions of pharmacological, immunological, and metabolic means and provides some useful examples to clarify these terms in the context of identifying the products’ principal mode of action (MoA). MoA is described in the guidance as the means by which the product achieves its main intended effect; it must be determined objectively using the most current scientific evidence. According to the regulatory framework, the pharmacological MoA focuses only on the initial interaction (binding) without explicitly mentioning signal transduction [7,8].

**Figure 1:**
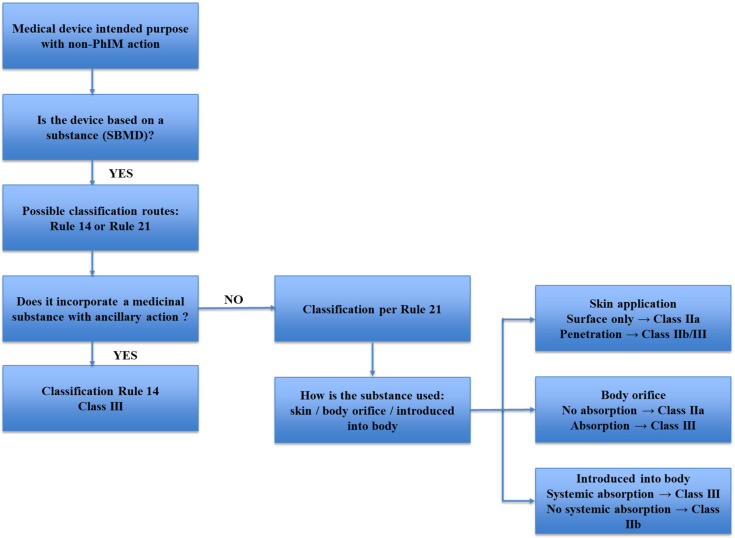
Decision flowchart for the classification of substance-based medical devices in accordance with Regulation (EU) 2017/745, Annex VIII, Rules 21 and 14. The diagram illustrates the stepwise assessment of the route of administration, degree of absorption and presence of medicinal substances with ancillary action, supporting the determination of the applicable risk class and conformity assessment pathway.

Substance-based medical devices (SBMDs) fall within this definition when their therapeutic effect is primarily driven by physicochemical mechanisms, such as barrier formation, lubrication, hydration, adsorption, or modulation of the local microenvironment. SBMDs fall within this definition when their therapeutic effect is primarily driven by physicochemical mechanisms, such as barrier formation, lubrication, hydration, adsorption, or modulation of the local microenvironment. In this context, the regulatory qualification of SBMDs does not depend solely on their composition or formulation but on the identification of the principal mode of action through which the intended medical effect is achieved. Consequently, the presence of medicinal substances with pharmacological properties does not preclude classification as a medical device, provided that such activity remains ancillary and does not constitute the principal intended action of the product, according to Annex VIII, Rule 14 or Rule 21 (Figure 1).

Rule 14 of the MDR specifically addresses MDs incorporating medicinal substances with an ancillary function, i.e., substances that support the medical device in achieving its intended purpose without exerting the principal mode of action: “*All devices incorporating, as an integral part, a substance which, if used separately, can be considered to be a medicinal product, as defined in point 2 of Article 1 of Directive 2001/83/EC, including a medicinal product derived from human blood or human plasma, as defined in point 10 of Article 1 of that Directive, and that has an action ancillary to that of the devices, are classified as class III*.” [2,7].

Compared to the previous regulatory framework, Directive 93/42/EEC on Medical Devices (MDD), the MDR introduced a more explicit focus, omitting the phrase “*liable to act*”, on the relationship between the device’s principal intended action and the role of the incorporated substance, thereby reinforcing the importance of a robust scientific justification of the ancillary nature of any pharmacological activity but consequently raising concerns among industry stakeholders and Notified Bodies [2,7].

**Figure 2:**
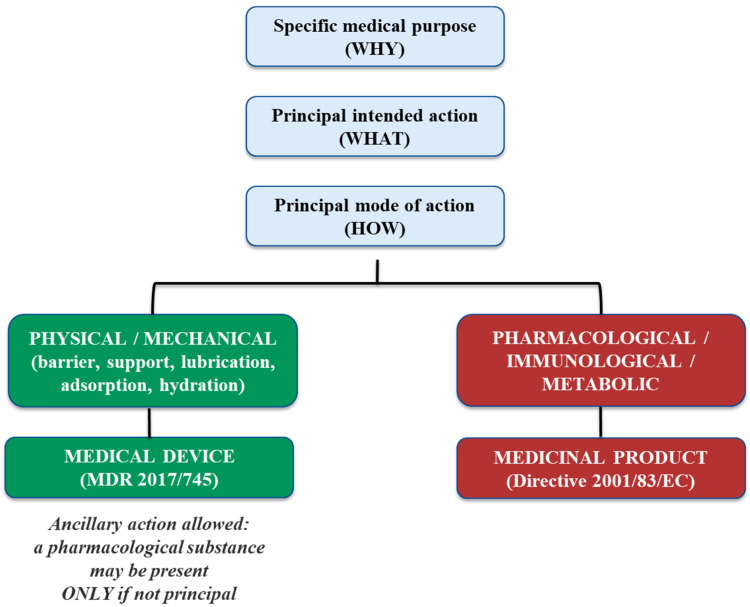
Conceptual decision framework for the determination of the Principal Mode of Action (PMOA) in accordance with Regulation (EU) 2017/745 and MDCG 2022-5 Rev.1. The diagram illustrates the hierarchical distinction between specific medical purpose (WHY), principal intended action (WHAT) and principal mode of action (HOW), highlighting the PMOA as the determining criterion for regulatory qualification of borderline products.

In this context, the determination of whether a substance exerts an ancillary or principal action is closely linked to the identification of the product’s principal MoA (Figure 2). As clarified by the MDCG 2022-5 Rev.1, the presence of a substance with pharmacological properties does not automatically trigger classification as a medicinal product, provided that such activity is secondary and not essential for achieving the intended medical effect of the device. The regulatory assessment must therefore be based on objective scientific evidence demonstrating that the primary therapeutic effect is achieved through physical or physicochemical mechanisms, with the medicinal substance acting solely in a supportive role. This clarification broadened the scope of Rule 14 to include cases such as substances exerting effects in blood bags or wound exudates. For legacy devices previously certified under the MDD, re-consultations with Competent Authorities are required under MDCG 2020-12 to verify the continued compliance of the ancillary medicinal substance [9]. This process involves submitting updated documentation on the substance itself, its method of incorporation, manufacturing processes, and any design elements that could impact the substance’s quality, safety, or functionality within the device. The Notified Body must also provide a declaration regarding any changes affecting these aspects.

While Rule 21 outlines a risk-based framework based on the site of application and extent of absorption, as reported below: “*Devices that are composed of substances or of combinations of substances that are intended to be introduced into the human body* via *a body orifice or applied to the skin and that are absorbed by or locally dispersed in the human body are classified as:*

- *class III if they, or their products of metabolism, are systemically absorbed by the human body in order to achieve the intended purpose;*
- *class III if they achieve their intended purpose in the stomach or lower gastrointestinal tract and they, or their products of metabolism, are systemically absorbed by the human body;*
- *class IIa if they are applied to the skin or if they are applied in the nasal or oral cavity as far as the pharynx, and achieve their intended purpose on those cavities; and*
- *class IIb in all other cases.*”

According to the MDCG 2022-5 Rev.1, such devices typically act through mechanisms like physical barrier formation, lubrication, or modulation of the local microenvironment, distinguishing them from MPs whose action is pharmacological, immunological, or metabolic [7].

The application of Rule 21 requires a careful scientific assessment of the product’s physicochemical properties and mechanisms of action, particularly for topical formulations used in wound care. In this setting, SBMDs typically exert their intended effect through physical or physicochemical mechanisms, such as barrier formation, hydration, adsorption of exudate, modulation of the wound microenvironment, or control of microbial burden. However, the coexistence of such mechanisms with antimicrobial substances may complicate the distinction between physical action and pharmacological effect, especially when antimicrobial efficacy contributes significantly to the clinical performance of the device.

As a result, the interpretation of Rule 21 is closely interconnected with the identification of the principal mode of action and, where applicable, with the assessment of ancillary pharmacological activity under Rule 14 (Figure 1). Inconsistent interpretation of these criteria across Member States has contributed to divergent classification outcomes for similar SBMDs, underscoring the need for a harmonized, evidence-based application of Rule 21 aligned with MDCG guidance and scientific state of the art.

As emphasized by the Association of the European Self-Care Industry (AESGP), the application of Rule 21 must be based on a case-by-case assessment, considering both product composition and clinical context, including the use of substances with a well-established safety profile in food or cosmetics [10].

Before the new regulation, SBMDs were included in Directive 93/42 but were not clearly defined at the time. As a result, they were often considered “borderline” products, somewhere between medical devices and medicinal products, until the MDR was introduced [11].

SBMDs include devices made from chemically defined substances as well as those derived from biological sources. Although the MDR still does not provide a formal definition for them, it offers detailed rules for their regulation. These are found in General Safety and Performance Requirements (GSPRs) in Annex I of the MDR (e.g., 12.2, 13.3, 23.2(r), 23.4(t)) and a dedicated certification process (Annex IX, Section 5.4) [12].

### Topical Substance-Based Medical Devices

Topically applied SBMDs constitute an increasingly relevant therapeutic category whose regulatory qualification under the MDR primarily hinges on the demonstration of a non-pharmacological principal mode of action, distinguishing them from topical medicinal products (Figure 2) [13]. SBMDs have been successfully employed across various therapeutic areas, notably dermatology, wound care, mucosal protection, and gastrointestinal disorders. In dermatology, topical SBMDs address skin conditions such as eczema, psoriasis, and dermatitis by reinforcing barrier integrity and hydration. In wound care, these products facilitate wound healing through the physical protection of tissues and maintenance of an optimal microenvironment, thus supporting natural repair mechanisms. Furthermore, SBMDs play an essential role in mucosal protection, alleviating symptoms of irritation or inflammation by creating a soothing protective layer over mucous membranes [13,14,15]. These topical SBMDs commonly act through physical or physicochemical means such as the formation of protective barriers, lubrication, hydration, or adsorption onto biological tissues, facilitating conditions optimal for healing or symptom relief related to gastrointestinal disorders [4]. Additionally, they may function through adsorption or mucoadhesion, ensuring the retention of active substances at the target site, thus enhancing local efficacy without systemic absorption [14,16]. Other examples include osmotic effects, viscosity modification, and modulation of local pH, further distinguishing these devices from topical pharmaceuticals and cosmetics [15].

Under Regulation (EU) 2017/745, Annex VIII, Rule 21 formally establishes a risk-based classification framework for topical SBMDs, whereby regulatory qualification is driven by the degree of absorption and intended site of action, with significant implications for evidentiary requirements related to safety, biocompatibility, and clinical performance (Figure 1).

## 3. Market Dynamics

According to recent industry reports, the European medical technology sector was valued at approximately €160 billion in 2023, with a sustained annual growth rate of 5–6% over the previous decade [17]. Market data from five European countries (Italy, Spain, France, Poland, and Germany) highlight the growing relevance of substance-based medical devices (SBMDs) in the European self-medication sector. Based on data mainly from IQVIA, the combined market value of SBMDs in these countries reached approximately €3.2 billion, representing 304 million units sold as of mid-2022 (Source: IQVIA Flexview Multichannel Italia—MKT Moving Annual Total Apr 2022; [13]). This corresponds to a growth rate of +18%, outperforming the overall self-medication sector, which grew by +13.5%. These devices now account for 11% of the self-medication market, with an average price per unit of €10.41, compared to €7.47 for the total self-medication sector, suggesting a perceived higher value and innovation level. Italy, considered a benchmark market, demonstrates particularly strong figures: in April 2022, over 650 companies were active in the SBMDs sector, generating €1.1 billion in sales. SBMDs, especially those derived from natural substances, are becoming a core element of the EU health ecosystem. Their success reflects both market demand and the ability of the newest MDR framework to support innovation, safety, and therapeutic diversity in addressing unmet medical needs [13]. Such dynamic growth highlights consumer preference for innovative, multifunctional topical products, particularly in therapeutic areas traditionally dominated by medicinal products, such as dermatology and wound care.

The historical evolution and regulatory developments underscore a notable shift towards sophisticated topical delivery systems, driving significant investment and innovation. Indeed, the rise of SBMDs illustrates the interplay between consumer demand, scientific advancement, and regulatory clarity. Their emergence not only reshapes the topical therapeutic landscape but also challenges existing classifications, necessitating continuous refinement of regulatory frameworks to accommodate innovation without compromising safety and effectiveness [13].

## 4. Topical Medicinal Products

Directive 2001/83 defines MPs as “*any substance or combination of substances*” intended to exert a therapeutic effect by modifying physiological functions through a pharmacological, immunological, or metabolic mechanism of action [18].

These products typically involve active pharmaceutical ingredients (API) that interact with skin or mucosal receptors or enzymes to modulate physiological processes. A clear example is topical corticosteroids, which act as glucocorticoid receptor agonists, regulating gene expression to suppress inflammation and immune activity [16,19]. The clinical use of corticosteroids for inflammatory dermatoses like eczema and psoriasis demands careful benefit-risk evaluation, especially in terms of potency and duration of use, to minimize systemic side effects such as hypothalamic–pituitary–adrenal axis suppression and skin atrophy [20].

Because their primary action is pharmacological, topical MPs are regulated under Directive 2001/83/EC, which mandates a rigorous marketing authorization process that involves EMA. This includes submission of comprehensive quality, safety, and efficacy data, derived from nonclinical and clinical trials, to demonstrate a favorable benefit-risk profile [18]. Randomized controlled trials (RCTs) offer the highest level of clinical evidence; for instance, combination therapies such as lebrikizumab plus topical corticosteroids have undergone robust RCT evaluation for atopic dermatitis, focusing not only on efficacy but also on safety endpoints [21].

In contrast to SBMDs, which act through physical or chemical means, topical MPs inherently rely on the biological activity of APIs. This reliance translates into stricter pre-market requirements, including evidence of pharmacokinetics, systemic exposure, and long-term toxicity. For example, guideline-driven clinical evaluations must address efficacy endpoints, adverse event profiles, and risk mitigation strategies (e.g., potency limitation, treatment duration controls) to satisfy regulatory obligations under the EMA Committee for Medicinal Products for Human Use (CHMP) and national authorities [22]. As mentioned, SBMDs, which may contain a medicinal product as an ancillary substance, differ both technically and regulatorily from pure MPs. While the ancillary substance may contribute to the overall performance, its action must remain secondary to the non-pharmacological mechanism of the device. Consequently, these products are assessed under the MDR framework, with involvement of a Notified Body and a mandatory consultation with EMA or national competent authorities for the ancillary substance. Unlike medicinal products, where full pharmacokinetic, systemic exposure, and efficacy data are required, SBMDs focus on demonstrating the safety, quality, and compatibility of the ancillary component and on ensuring that it does not pose disproportionate risks. Clinical evaluation therefore aims at confirming safety and performance of the device, while the ancillary substance is scrutinized mainly for safety and supportive contribution, rather than as the primary determinant of therapeutic efficacy.

## 5. Innovations in Wound Dressings: From Traditional Approaches to SBMDs

Traditional wound care strategies include a variety of approaches, ranging from non-invasive therapies and pharmacological treatments to surgical interventions. Among non-invasive methods, topical formulations, wound dressings, and skin substitutes are widely used [23]. Wound dressings play a central role in both acute and chronic wound management by maintaining a favorable microenvironment that supports tissue regeneration and reduces the risk of infection. In recent years, advances in materials science, biotechnology, and nanotechnology have led to the development of a wide range of innovative dressings tailored to different wound types and clinical needs [23].

Currently, more than 3000 wound dressing products are available on the market, generally classified as traditional, biological, or synthetic, depending on their composition and mechanism of action. The most used are film-based, hydrogel-based, and polymer-based formulations, often incorporating antimicrobial agents, particularly for the treatment of acute or infected wounds [24]. Conventional dressings such as gauze, cotton pads, and bandages primarily act as passive barriers, offering limited biological support. In contrast, modern dressings, such as hydrogels, hydrocolloids, alginates, foams, and films, are specifically designed to preserve the wound microenvironment by maintaining moisture, temperature, and pH balance, thus promoting re-epithelialization, protecting surrounding tissues, and reducing bacterial colonization [24,25,26].

An ideal wound dressing should meet multiple criteria: it should ensure a moist environment; allow gas exchange; conform to the wound bed; absorb excess exudate; promote cell migration and proliferation (key for angiogenesis and tissue regeneration); provide hemostatic control; maintain thermal insulation; prevent bacterial contamination; be occlusive yet non-adherent; exhibit biocompatibility, non-toxicity, and mechanical stability; allow atraumatic removal; facilitate debridement; minimize scar formation; and be readily available and cost-effective [24].

Driven by these complex requirements, research has increasingly focused on dressings based on biopolymers and bioactive compounds, formulated in novel pharmaceutical-like formats such as hydrogels, scaffolds, fibers, sponges, membranes, and films. Common biopolymers, whose advantages and disadvantages are described in Table 1, used in these formulations include chitosan, polyvinyl alcohol, cellulose, polycaprolactone, gelatin, starch, collagen, hyaluronic acid, alginate, polylactic acid and carrageenan. Notably, chitosan, a natural cationic polysaccharide derived from chitin, has demonstrated excellent antimicrobial properties, biocompatibility, biodegradability, non-toxicity, and wound-healing capabilities [25,27,28]. Due to the presence of amine groups, it becomes positively charged at pH values below 6. This positive charge enables chitosan to electrostatically interact with negatively charged sites on microbial membranes. As a result, chitosan can attach to the bacterial cell surface and disrupt membrane integrity, leading to the leakage of intracellular contents and the inhibition of nutrient transport into the cell [29,30]. An injectable hydrogel composed of two natural polymers, chitosan and konjac glucomannan, crosslinked through Schiff base bonds, demonstrated strong antibacterial activity against both Gram-positive *Staphylococcus aureus* and Gram-negative *Escherichia coli*, achieving killing efficiencies of 96% and 98%, respectively [31]. In another approach, chitosan/bacterial cellulose semi-interpenetrating hydrogels were prepared by blending the two polymers, followed by crosslinking with glutaraldehyde. These hydrogels also exhibited antibacterial activity against the same bacterial strains, with the level of antibacterial effectiveness depending on the chitosan-to-cellulose ratio [32].

Another important example is hyaluronic acid (HA), a naturally occurring polysaccharide extensively used in hydrogel-based dressings due to its high hydrophilicity, biocompatibility, and ability to retain moisture and support tissue repair [33,34,35]. Topical dressings that combine HA with antimicrobial agents such as metallic silver or silver sulfadiazine (SSD) are among the most representative examples of substance-based medical devices (SBMDs). These formulations blur the regulatory line between medical devices and medicinal products. In particular, HA/AgNPs (silver nanoparticle) nanocomposites have shown strong antibacterial activity against Gram-negative bacteria such as *Escherichia coli*, with high biocompatibility and no cytotoxic effects on keratinocyte cell lines. Histological analyses confirmed enhanced wound healing compared to HA-only or untreated controls [36]. The natural biocompatibility of hydrogels also enables the incorporation of small active molecules, such as antibiotics, which can exert bacteriostatic or bactericidal effects through concentration-dependent mechanisms targeting microbial structures and metabolic processes [27].

Among antimicrobial agents reported in Table 2, silver has been used for centuries in infection control and remains a cornerstone in modern wound care. Its broad-spectrum activity covers both Gram-positive and Gram-negative bacteria, making it suitable for managing heavily infected or drug-resistant wounds [37,38]. Silver ions act on multiple bacterial targets, including membranes, cytoplasmic organelles, and DNA, which makes resistance development rare. Silver is often embedded in advanced dressing materials like foams, alginates, hydrogels, and hydrofibers, enabling sustained release in moist environments and effective exudate management while preventing periwound maceration [39]. Silver nanoparticles are typically incorporated at 0.05–0.5 wt% relative to the dry polymer matrix (i.e., 500–5000 ppm), a range chosen to provide sufficient antibacterial activity while limiting cytotoxicity, with final optimization based on silver ion release and cell viability testing [40,41].

Silver sulfadiazine (SSD), synthesized from silver nitrate and sulfadiazine, has been a standard topical antimicrobial since the 1970s, particularly for burn wounds. Its dual-action mechanism, combining the antimicrobial effects of silver and the bacteriostatic activity of sulfadiazine, has proven superior to other topical antiseptics like PVP-I [42]. Silver sulfadiazine is generally incorporated at around 1 wt% of the gel or polymer matrix, within the established 1% *w*/*w* range used in SSD hydrogels and topical burn preparations, with possible exploration up to 2.5–5% *w*/*w* in experimental studies if justified by safety data [43,44,45]. However, challenges related to its solubility and stability have limited its broader application in controlled drug delivery systems. In response, silver nanoparticles (AgNPs) have gained attention as a next-generation alternative, demonstrating effective biofilm disruption, membrane damage, and enhanced cell migration in wound models. Their integration into collagen- or chitosan-based scaffolds further improves healing performance [46].

In this context, Rule 14 of the MDR is particularly relevant, as it applies to medical devices that incorporate, as an integral part, a substance that, if used separately, would be considered a medicinal product according to Directive 2001/83/EC, and whose action is ancillary to that of the device. This rule is especially important when assessing wound dressings containing SSD or silver nanoparticles.

According to the AESGP Position Paper on Rule 14 (2018), the classification of such products requires a case-by-case assessment that considers not only the presence and amount of the active substance but also whether it is capable of appreciably restoring, correcting, or modifying physiological functions through a pharmacological, immunological, or metabolic (PhIM) action. If these conditions are met and the action remains ancillary, the product falls under Rule 14 and is classified as a Class III medical device. However, if the PhIM action is deemed primary, the product must be regulated as a medicinal product.

This distinction is critical for topical antimicrobial formulations, where the line between physical/chemical barrier function (typical of SBMDs under Rule 21) and active pharmacological antimicrobial action (indicative of a medicinal product) is often blurred. As the AESGP notes, scientific justification must support the manufacturer’s claim regarding the principal mode of action, and such claims cannot contradict current scientific evidence or pharmacological plausibility [47].

**Table 1:** Comparative table outlining the advantages and disadvantages of polymer matrices employed in wound dressings.

Polymer	Advantages	Disadvantages	References
Chitosan	Antimicrobial, hemostatic, biocompatible and biodegradable	Solubility limitations, mechanical constraints, heterogeneity and batch variability	[48,49,50,51,52]
Polyvinylalcohol	Biocompatible, water solubility, hemostatic, high-water uptake, low production costs	Non-antimicrobial, limited mechanical properties	[53,54,55,56,57,58,59]
Cellulose	Biocompatible, hemostatic, water absorption capacity	Non-antimicrobial, limited mechanical properties	[60,61,62,63]
Polycaprolactone	Biocompatible, mechanical strength, controllable degradation rate, cost-effectiveness and scalability	Non-antimicrobial, hydrophobicity, poor absorption capacity	[64,65,66,67]
Gelatin	Biocompatible, hemostatic, versatile formulation capability, water absorption capacity	Non-antimicrobial, limited mechanical properties, thermosensitive, batch variability, high production costs	[68,69,70,71]
Starch	Biocompatible, water absorption capacity, cost-effectiveness, versatile formulation capability, excellent water vapor transmission rate	Non-antimicrobial, limited mechanical properties, environmental sensitivity, batch variability, high production costs and scalability challenges	[72,73,74,75,76]
Collagen	Biocompatible, hemostatic, angiogenic, versatile formulation capability, biodegradable, promote fibroblast proliferation and collagen deposition	Non-antimicrobial, high production costs, manufacturing complexity, weak mechanical properties, immunogenicity and allergic sensitization, ethical/religious limitations	[77,78,79,80,81,82,83]
Hyaluronic acid	Biocompatible, anti-inflammatory, antioxidant, optimal moist wound management, angiogenic, versatile formulation capability	Weak mechanical properties, high production costs, batch variability, maceration risk for high swelling ratio, non-antimicrobial	[34,84,85,86,87,88,89,90,91]
Alginate	Biocompatible, exudate absorption, moisture management, hemostatic, anti-inflammatory, versatile formulation capability, cost-effectiveness	Weak mechanical properties, non-antimicrobial, rapid enzymatic degradation, complex production, high variability	[92,93,94,95,96,97,98,99]
Polylactic acid	Biocompatible, superior mechanical properties, promotes angiogenesis and collagen deposition, optimal moisture management, versatile formulation capability	Slow degradation rate, acid degradation by-products, inherent hydrophobicity, low cell affinity, non-antimicrobial, high production costs,	[100,101,102,103,104,105,106]
Carrageenan	Biocompatible, superior exudate absorption and moisture management, hemostatic, versatile formulation capability, antioxidant, anti-inflammatory	Weak mechanical properties, non-antimicrobial, sourcing and batch variability, high production costs, batch	[107,108,109,110,111,112,113]

**Table 2:** Comparative table of the advantages and disadvantages of various antimicrobial agents employed in wound dressings.

Antimicrobial Agents	Advantages	Disadvantages	References
Silver	Broad-spectrum antimicrobial, bactericidal, anti-inflammatory	Cytotoxicity, argyria and systemic silver accumulation	[114,115,116,117,118,119,120]
Metals and metal oxides nanoparticles (silver, zinc oxide, iron oxide, cerium dioxide, titanium dioxide)	Broad-spectrum antimicrobial, anti-inflammatory, antioxidant	Dispersion and accumulation in different organs of the body, leading to toxicity	[121,122,123,124,125]
Non-metal nanoparticles (dendrimers, ferritins, micelles, liposomes)	Broad-spectrum antimicrobial, low immunogenicity, and encapsulation capacity	High production costs	[38,126,127,128,129]
Iodine	Broad-spectrum antimicrobial, bactericidal, fungicidal, rapid efficacy, anti-inflammatory, cost-effectiveness,	Local tissue toxicity and irritation, thyroid dysfunction risk	[130,131,132,133,134,135,136]
Biguanides: polyhexamethylene biguanide (PHMB), chlorhexidine	Cationic emulsifier and broad-spectrum antimicrobial, bactericidal, virucidal, cysticidal, promotes tissue granulation and wound healing	Possibly cytotoxic, repeated prolonged exposure may cause sensitization	[38,137,138,139,140,141]
Plant-derived natural compounds (oregano, tea tree oil, lavender)	Broad-spectrum antimicrobial, bactericidal, insecticidal, analgesic, antioxidant, and anti-inflammatory effects	Frequent application and/or the use of high concentrations may be necessary, batch variability	[142,143,144,145,146,147,148]
Antimicrobial peptides (AMPs)	Broad-spectrum antimicrobial, biofilm penetration and disruption, minimal resistance development	Some AMPs might be sensitive to light, heat, and moisture; proper storage conditions are crucial to maintain their efficacy and cytotoxicity at higher concentrations. High production costs	[149,150,151,152,153,154]

## 6. Examples of Registered Topical Substance-Based Medical Devices

Examples of topical dressings, based on substances, available on the market are given in Table 3. Some of the products are briefly commented on below.

**Table 3:** Examples of topical dressings registered as SBMDs and available on the market; trade name, manufacturer, risk class and applicable legislation are mentioned.

Trade Name	Manufacturer	Risk Class	Applicable Legislation	Reference
Bioepithelia base crema	Kethema farmaceutici srl	Class I	Reg. UE 2017/745	[155]
Alovex ferite spray	Nirial pharma s.r.l.	Class IIa	Reg. UE 2017/745	[156]
Ialuset	Ibsa farmaceutici italia srl	Class IIb	Reg. UE 2017/745	[157]
Iodosorb ointment	Smith and nephew medical limited	Class III	Reg. UE 2017/745	[158]
Sofargen repair	Sofar s.p.a.	Class IIa	Directive 93/42/EEC on Medical Devices	[159]
Fitostimoline plus crema	Farmaceutici damor s.p.a.	Class IIb	Directive 93/42/EEC on Medical Devices	[160]
Connettivinabio plus crema	Fidia farmaceutici s.p.a.	Class III	Directive 93/42/EEC on Medical Devices	[161]
ialuset plus	Ibsa farmaceutici italia srl	Class III	Directive 93/42/EEC on Medical Devices	[162]

Taking into consideration Ialusetcare, for example, is a medical device indicated for the treatment of various skin lesions, including non-infected wounds, cuts, burns, leg ulcers, hand fissures, fistulas, pressure ulcers, abrasive dermatitis, scars, and excoriations. It is also recommended for moderate to severe skin irritation and dehydration, such as that occurring during radiotherapy. Additionally, the cream may be used to support re-epithelialization in difficult-to-heal wounds and major scars, including post-surgical ones, helping to restore the physiological condition of the skin [162]. Connettivina^®^ bio plus is recommended for managing skin lesions at high risk of infection. It is particularly suitable for both acute and chronic wounds, including first- and second-degree burns, vascular and metabolic ulcers, and pressure sores. This formulation helps maintain a moist, microbe-free wound environment [161]. Another product, Connettivinasilver^®^ Plus spray, is a topical powder spray composed of sodium hyaluronate and metallic silver. It is indicated for the temporary topical treatment of non-infected skin injuries such as abrasions, excoriations, fissures, cuts, minor surgical wounds, and localized first- and second-degree burns. The device promotes a humid wound environment while protecting the lesion from external microbial contamination [161].

All these medical devices are composed of substance-based formulations containing hyaluronic acid (HA), alone or in combination with silver-based antimicrobial agents, and share the same non-PhIM mechanism of action. HA, owing to its strong hygroscopic properties, plays a key role in maintaining a favorable microenvironment for wound healing by supporting skin hydration and elasticity in the presence of tissue damage. The hydrogel-based matrix, typically containing over 95% water, ensures continuous moisture at the wound site, thereby promoting the physiological healing process and facilitating the autolytic debridement of necrotic tissue.

In formulations that include silver sulfadiazine (SSD), a metal–organic compound obtained by reacting silver nitrate with sulfadiazine, an additional antimicrobial effect is provided, helping to prevent and control microbial contamination within the dressing and maintaining a wound environment free from exogenous pathogens. The overall formulation creates optimal conditions for tissue regeneration, acting as a physical barrier to microbial penetration while remaining easy to remove through gentle rinsing with saline solution.

In addition, several clinical trials were retrieved to support the safety and efficacy of wound healing through use of HA dressing. The study conducted by Gazzabin et al. (2019) evaluated the medical device Hyalosilver^®^, a topical spray powder containing HA and metallic silver [163]. The findings indicate that the spray is effective not only in reducing wound size from the first application but also in lowering the bacterial load. Quantitative analyses demonstrated a significant reduction in bacterial presence at both one and seven days after treatment, although complete eradication was not achieved in any of the cases [163]. The study by Dereure et al. (2012) was based on the hypothesis that the HA-containing formulation (Ialuset^®^ cream) would demonstrate superior clinical efficacy compared to its neutral vehicle (an identical formulation lacking HA) [164]. The primary endpoint, percentage reduction in wound size at day 45, was evaluated as a potential surrogate marker for leg ulcer healing [164]. The study by Barrois et al. (2007) evaluated the use of Ialuset^®^ cream (a 0.2% hyaluronic acid-based topical formulation) and Ialuset^®^ gauze pads (containing 0.05% hyaluronic acid) in the treatment of pressure ulcers [165]. Over a three-week period, complete wound healing was observed in only 1 out of 20 patients, suggesting that extended treatment durations and follow-up are necessary to fully assess the therapeutic potential of Ialuset^®^. Additionally, the study reported a significant reduction in the mean percentage of fibrous tissue within the wounds, along with a near-significant increase in granulation tissue [165]. In a single-center, observational, and retrospective study conducted by De Francesco et al. (2022) involving eighty patients, both cream and gauze formulations containing low molecular weight HA (200 kDa) and 1% SSD were well tolerated [166]. Most participants reported high levels of satisfaction with the treatments. The findings underscore the value and efficacy of appropriately designed wound dressings, such as Connettivina^®^ Bio Plus cream and gauze pads. The presence of LMW hyaluronic acid was shown to promote granulation tissue formation, offer protection against bacterial biofilms, and improve the condition of the perilesional skin. The observed enhancement in ulcer healing appears to result from the synergistic effect of HA and SSD. HA contributes to the creation of a moist wound environment that supports cell viability, tissue regeneration, and fluid retention, key factors in granulation tissue development and subsequent wound closure.

In combination, SSD adds further therapeutic benefit by inhibiting bacterial proliferation and reducing infection risk. The study demonstrated a strong correlation between bacterial control, effective wound bed management, and improvement of the periwound area, an essential aspect in the treatment of chronic ulcers. Overall, the treatment was associated with a notable reduction in discomfort, erythema, and edema [166]. The study conducted by Russo et al. (2022) aimed to evaluate and compare the efficacy and tolerability of two advanced wound dressings, Fitostimoline^®^ Plus and Connettivina^®^ Bio Plus, in the management of acute superficial skin lesions; both treatment groups demonstrated efficacy in reducing fibrin within the lesion [167]. These results align with existing literature, which consistently demonstrates that fibrin reduction facilitates the removal of a degraded extracellular matrix and supports the formation of a functional provisional matrix, thereby promoting a more physiological wound healing process [167].

## 7. Conclusions

The growing interest in SBMDs for topical wound care reflects a broader trend in biomedical innovation: the convergence of pharmaceutical-like formulations with medical device principles. Products containing biopolymers such as hyaluronic acid and chitosan, often combined with silver-based agents (e.g., silver sulfadiazine, AgNPs), demonstrate significant therapeutic potential while relying on physical or physicochemical mechanisms of action, a hallmark of SBMDs under Rule 21 of the MDR.

However, the regulatory classification of these products remains highly complex. The boundary between SBMDs under Rule 14 and MPs is often blurred, particularly when antimicrobial substances are incorporated. As highlighted in MDCG 2022-5 Rev.1 and reaffirmed by the AESGP position paper, a thorough, evidence-based assessment of the principal mode of action is essential. Yet, interpretations vary across Member States, creating uncertainty for developers and inconsistencies in market access.

The paradox is clear: a product classification may depend less on its clinical performance than on the regulatory lens through which it is viewed. A cream that hydrates and protects the wound is a device until it also claims to “kill bacteria,” at which point it may be seen as a drug. This binary logic, rooted in the separation between pharmacological and physical mechanisms, no longer reflects the complexity of modern therapeutic products, particularly those based on natural or multifunctional substances.

To foster innovation without compromising safety, a shift is needed from rigid categorization toward a more integrated and harmonized regulatory approach. This includes clearer guidance on the role of ancillary substances (Rule 14), greater acceptance of complex modes of action, and consistent application of borderline classification principles across Europe.

In conclusion, topical SBMDs represent not just a therapeutic opportunity but a regulatory challenge that forces us to rethink how we define, evaluate, and legitimize medical technologies. After all, a dressing should not become a drug merely because it works well, and the future of wound care depends on our ability to recognize that.

## 8. Key Takeaways

- Topical substance-based medical devices offer significant therapeutic potential in wound care, particularly when formulated with hydrating biopolymers (e.g., hyaluronic acid) and antimicrobial agents (e.g., silver sulfadiazine, AgNPs).
- The principal mode of action is the defining criterion for classification under MDR, impacting whether a product is regulated as a medical device (Rule 21), a drug-device combination (Rule 14), or a medicinal product.
- Products containing silver or SSD exemplify borderline challenges, as their antimicrobial activity may be ancillary (Rule 14) or primary (MPs), depending on concentration, presentation, and intended purpose.
- The interpretation of Rule 14 has been clarified by MDCG 2022-5 Rev.1 and the AESGP Position Paper, which stress the need for a case-by-case assessment and scientific justification of the mode of action.
- Inconsistent classification practices across EU Member States delay market access, increase regulatory burden, and hinder innovation, particularly for complex, multifunctional, or natural-origin substances.
- A more harmonized, flexible, and evidence-based regulatory approach is urgently needed to support the development of effective, safe, and innovative wound care solutions.

## Data Availability

No new data were created or analyzed in this study.
